# Bioelectrical impedance vector analysis (BIVA) to evaluate seasonal variations in body composition of elite soccer players

**DOI:** 10.1186/1550-2783-8-S1-P37

**Published:** 2011-11-07

**Authors:** Angelini Bonuccelli, Fabrizio Marzatico, Gianluca Stesina, Luca Stefanini, Daniela Buonocore, Sara Rucci, Fabrizio Tencone, Luca Gatteschi, Fabrizio Angelini

**Affiliations:** 1 Society of Sport Nutrition and Wellness; 2 Medical Staff, Juventus FC, Turin, Italy; 3 Laboratory of Pharmacobiochemistry, Nutrition and Nutriceuticals of Health, University of Pavia, Pavia, Italy; 4 Italian Football Federation Medical Department, Coverciano, Florence, Italy

## Background

The body composition and its variation in time can affect the performances of soccer players. The body composition measuring techniques are based on a quantitative approach founded on indirect estimations of fat mass and lean body mass. The BIVA allows us to directly see the athlete's body composition by means of impedance vector measuring (Z vector), irrespective of weigh and body hydration status. The BIVA can classify hydration and mass variations of the soft tissues and recent observations testify that the higher the soccer player’s level, the larger the quantity of soft tissue. The purpose of this study is to observe the season variations of the soft tissues, as an indirect estimation of the nutritional condition of Italian Serie A elite male soccer players.

## Methods

Resistance and reactance of the impedance vector (Z vector) were measured at 50 kHz (BIA 101 RJL, Akern Bioresearch, Florence, Italy) for a total of 18 players 27.6 ± 4.9 of age (Average ± DS) during a whole season. Inactive players, due to injury, were not tested. Tests were performed at the beginning(T0), at the end of the preseason training (T1), and afterwards every month (T2-T10) till the end of the championship. Eleven measurements were performed in total.

## Results

The position of the average impedance vector significantly diverged (Hotelling T2 test, p < 0.001), indicating a more favourable condition of the soft tissues (hydration and/or mass) in the subsequent months: a) T1, T3-T6 e T10 in respect to T0; b) T2, T8 e T10 in respect to T3; c) T10 in respect to T5; d) T10 in respect to T8.

## Conclusion

The BIVA seems to be a promising and useful means of body composition analysis for elite soccer players, at least in terms of variation of soft tissues (mass and hydration).

